# Oral Health Status of Children in Strasbourg: An Epidemiological Study (2018–2022)

**DOI:** 10.3390/children13010004

**Published:** 2025-12-19

**Authors:** Damien Offner, Hayat Heddoub, Sabine Chemouni, Gabriel Fernandez de Grado

**Affiliations:** 1 Faculté de Chirurgie Dentaire, Université de Strasbourg, 8 rue Ste Elisabeth, F-67000 Strasbourg, France; 2 Hôpitaux Universitaires de Strasbourg, 1 Place de l’Hôpital, F-67000 Strasbourg, France; 3 French National Institute of Health and Medical Research (INSERM), UMR 1260, Regenerative Nanomedicine (RNM), 1 Rue Eugène Boeckel, F-67000 Strasbourg, France; 4 Centre de Santé Dentaire, Ville et Communauté Urbaine, F-67000 Strasbourg, France

**Keywords:** child oral health, carious disease, oral epidemiology, oral prevention

## Abstract

**Highlights:**

**What are the main findings?**

• Children participating in a school-based oral health prevention and screening program show progressive improvement in their oral health throughout their school years.
• Children’s oral health seems to have been deteriorating for several years. Socioeconomic inequalities remain significant.

**What are the implications of the main findings?**

• National programs should be implemented to prevent the deterioration of the children’s oral health and to gather data since no national study was performed since 2006.
• Prevention programs in schools combining primary, secondary and tertiary prevention should be implemented.

**Abstract:**

Background/Objectives: Dental caries prevalence remains high in France, but data are scarce. In Strasbourg, a local program aims at improving oral health of all school children, from primary to tertiary prevention. We evaluated the oral health of Strasbourg’s children over five repeated cross-sectional screenings. Methods: We analyzed 58 287 screenings of children from 1st to 5th grade from 2018 to 2022. The presence and number of carious lesions was the main variable studied. Results: Untreated dental caries were present among 34% of the children and slightly increased from 33% in 2018 to 35% in 2022 (*p* = 0.002). The mean number of decayed teeth was 0.85 ± 1.68 and 2.5 ± 2.04 among children with at least one. Dental caries was more prevalent among children in schools from socially disadvantaged neighborhoods (43% vs. 27%, *p* < 0.001), children from less advanced classes (27% in 5th grade vs. 37% in 1st grade, *p* < 0.001), and children with inadequate hygiene (72% vs. 28%, *p* < 0.001). The number of decayed teeth followed the same trend as the prevalence. Conclusion: The prevalence of dental caries diminishes throughout the schooling years. Socioeconomic and behavioral factors remain the major predictors of dental caries. A national study could help confirm if these trends are widespread in France.

## 1. Introduction

Dental caries is a chronic infectious disease with a multifactorial etiology, affecting dental tissues and leading to tooth destruction [1]. It is one of the most common non-communicable diseases worldwide, with a prevalence of approximately 3.5 billion people. Its burden is increasing, especially in low- and middle-income countries. Dental caries is mostly caused by the dietary and oral hygiene behaviors of the patient, thus being strongly linked to socioeconomic inequalities [2].

Nowadays, dental caries represents a public health issue due to its high prevalence and incidence, as well as its impact on general health [3]. Trends over recent decades have shown improvements in oral health, although dental caries and its complications persist [4].

In France, the direct costs of dental care are estimated at EUR 11.1 billion, with indirect costs at EUR 6.3 billion, and the prevalence of oral diseases approaches 100% according to the WHO [5]. It is therefore important to promote primary prevention through oral hygiene, of which tooth brushing is the principal but not the only element. It is an effective, low-cost, and easily implementable practice that can prevent almost all oral diseases, especially caries and gingivitis [4]. Secondary prevention, through early caries management, allows for the use of less invasive and less expensive conservative treatments, as opposed to the prosthetic treatments required for more advanced carious lesions.

The prevalence of caries in children seems to remain higher in France compared to many other countries, although this information should be interpreted with caution due to the lack of recent epidemiological studies on the topic [6,7]. The most recent national study dates back to 2006 [8]. The prevention of oral diseases is an ongoing issue that requires continuous attention and action.

Established in 1902, the Strasbourg Dental Health Center employs salaried dentists whose missions include collective oral health education sessions, annual systematic individual screenings in all public elementary schools in Strasbourg (France), and dental care during school hours in certain neighborhoods where children do not have a private practitioner. Overall, primary, secondary, and tertiary prevention are covered. Within a healthcare system that still favors curative care over preventive measures, this unique (due to its scope, the time it has lasted, and its systematization) initiative in France ensures the prevention of oral diseases at all levels, reaching the entire population of school-aged children in the public sector. A portion of this population is less receptive to volunteer-based prevention programs, such as the M’T Dents oral health check-ups, which previously invited children aged 3 to 24 to undergo screenings every three years and are now being annualized [9], and is thus more likely to benefit from Strasbourg’s systematic screening.

The objective of our study is to assess the oral health status of school-aged children in Strasbourg from 2018 to 2022, and to evaluate the state of dental caries, accounting for various determinants such as age, sex, and geographic location for the same period.

Our null hypothesis is that there was no significant change in the prevalence of dental caries from 2018 to 2022.

## 2. Methods

We analyzed data from the individual screenings described in the Introduction, which can be considered as five annual descriptive cross-sectional studies with repeated measures on the oral health status and associated risk factors among school children in Strasbourg.

### 2.1. Population/Eligibility Criteria

This study included all children screened during routine oral health check-ups conducted in primary schools across the city of Strasbourg. A representative sample of 58,287 children (female: 29,696; male: 28,590), aged 6 to 12 years, was examined between 2018 and 2022. The participants were all children enrolled in public primary schools in Strasbourg who provided their assent to participate in the screening program, with informed consent obtained from their parents. The screenings were conducted in 58 primary schools.

Some children were observed multiple times across different years. For the sake of clarity, we will keep using the term “children” throughout this article, even though each observation corresponds to a screening event, and the actual number of individual children is slightly lower, as some were examined multiple times over the 5 years of data collection, which may influence our results. Appropriate statistical methods were used (see below).

### 2.2. Data Collection

Screenings were conducted during school hours by a team consisting of 2 dentists, dental assistants, and sixth-year dental students from the Faculty of Dentistry at the University of Strasbourg.

The examinations were carried out by the 2 dentists using lamps, mirrors, and probes. Data were collected in a standardized manner using a checklist during the screening sessions, then entered into the patients’ electronic medical records, before being extracted and anonymized for analysis.

Data were collected for medical reasons. We worked on the already existing data for this study and could not add any variable that was not already measured.

The following variables were collected and analyzed:

• Demographic data: age and sex.
• Year of data collection.
• Geographical and school-related data: grade level, school name, district of the school (not analyzed in this article considering how hard it would be to interpret for people not familiar with Strasbourg’s geography), and school within the “Priority Education Network” (“Réseau d’Education Prioritaire”) with REP or REP+ status, i.e., schools cumulating unfavorable social determinants, especially for REP+. The REP status has been linked to a higher caries risk [10].
• Carious status: caries-free, treated (with no active caries), or presenting untreated carious lesions. The term “decayed tooth” will be used to refer to a tooth with an untreated carious lesion.
• Number of decayed teeth, when applicable.
• Oral hygiene assessment according to the investigator: adequate or requiring improvement (inadequate).
• Other required dental care: sealants and/or scaling.
• Children anonymous ID: required for the mixed-effect logistic regression.

### 2.3. Statistical Methods

Statistical analyses were performed using Stata/SE 13.0. For all statistical tests, the alpha risk was set at 0.05 and the beta risk at 0.20.

Proportions were compared using Pearson’s Chi-square tests. For the only quantitative variable—the number of decayed teeth—comparisons between groups were conducted using the Kruskal–Wallis test.

We used a mixed-effect logistic regression model with the presence of at least one decayed tooth as the dependent variable and including a random intercept per individual to account for the fact that some children were observed multiple times across different years.

## 3. Results

### 3.1. Population

From 2018 to 2022, 62,474 screenings were planned, and 58,287 children (93%) were examined. The sample consisted of 29,697 boys (51%) and 28,590 girls (49%). The mean age was 8.8 years, with most children between 6 and 10 years old. The distribution across grade levels was approximately uniform, with around 20% of participants in each grade from CP to CM2 (i.e., 1st grade to 5th grade).

### 3.2. Univariate Analysis

Half of the children had never experienced tooth decay (50%), 16% had received dental treatment but had no untreated carious lesion, and 34% had at least one decayed tooth. Most children (86%) had adequate oral hygiene, and only 9% required scaling.

**Figure 1:**
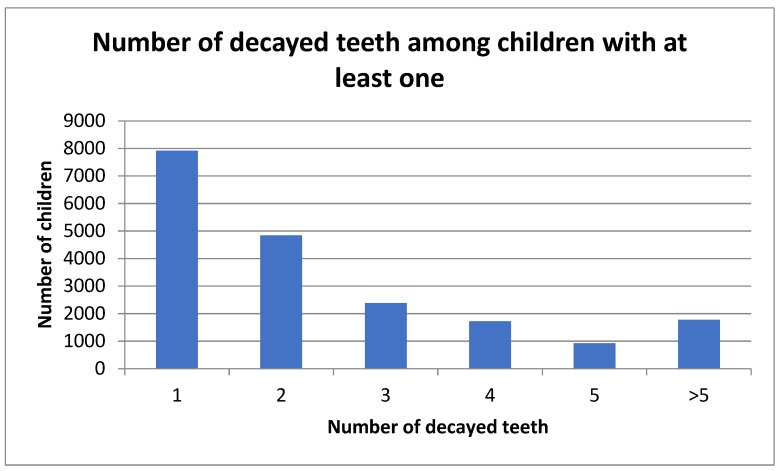
Distribution of the population according to the number of decayed teeth among children with at least one decayed tooth.

Among children with at least one decayed tooth, most had one (40%) or two (25%) decayed teeth (Figure 1). The number of decayed teeth per child followed an exponential decrease. Only a small proportion of children presented more than five untreated carious lesions, with a maximum of 18 untreated decayed teeth observed in a single child within the sample.

### 3.3. Bivariate Analysis

Inadequate oral hygiene was the main predictor associated with the presence of decayed teeth. Among children with inadequate oral hygiene, 72% had at least one decayed tooth compared to 28% of those with adequate hygiene. The mean number of decayed teeth was significantly higher among children with inadequate hygiene (Table 1).

Boys were more likely to present at least one decayed tooth than girls (36% vs. 32.7%, *p* < 0.001). They also displayed more decayed teeth. Girls were more likely to have adequate oral hygiene (87% vs. 84%, *p* < 0.001).

Children older or in higher grade levels were less likely to have decayed teeth and had fewer decayed teeth, if any.

**Figure 2:**
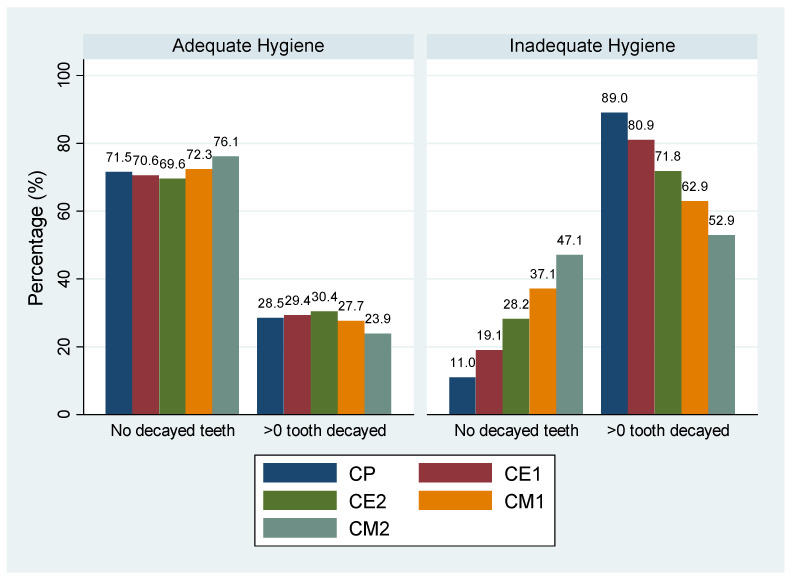
Percentage of children with at least one decayed tooth according to oral hygiene and grade.

An interaction between grade and adequate oral hygiene was found. Children with adequate oral hygiene had an almost stable prevalence of dental caries while children with inadequate oral hygiene showed a clear diminution of the frequency of dental caries as they reached higher grades (Figure 2).

Children from schools within the Priority Education Network (REP or REP+) were more likely to have decayed teeth (43% vs. 27%, *p* < 0.001) and had a higher number of affected teeth. No significant difference was observed between REP and REP+ schools. Children from REP/REP+ schools were also slightly less likely to have adequate oral hygiene (81% and 82% vs. 89%, *p* < 0.001).

The proportion of children with at least one decayed tooth slightly increased in 2022 compared to 2018 (35% vs. 33%, *p* = 0.002). The highest prevalence was observed in 2020 (37%), exceeding all other studied years.

### 3.4. Multivariate Analysis

A mixed-effect logistic regression model was used, with the presence of at least one decayed tooth as the dependent variable as described in Methods. All measured variables had a significant effect on this outcome and were therefore included in the analysis. Grade level was included in the model but not age, due to a strong collinearity between the two variables.

In the full model without interaction terms, having inadequate oral hygiene was the main predictive factor for the presence of decayed teeth, with an odds ratio (OR) of 9.31 (*p* < 0.0001). However, a significant interaction was observed between oral hygiene and grade level; the final model therefore included this interaction (Table 2). No other significant interaction was observed. The model was not significantly improved by adding schools as a random effect (to check for clustering), so the variable was not included. The model showed no sign of overfitting.

Among children with adequate oral hygiene, the probability of having at least one decayed tooth remained relatively stable across grade levels but decreased significantly in CM2 (fifth grade). Among children with inadequate hygiene, this probability was substantially higher than in those with adequate hygiene (OR = 44.66, *p* < 0.001 in CP [first grade]), but the difference gradually diminished as the prevalence of untreated caries decreased in the higher grades.

## 4. Discussion

### 4.1. Key Results

Our sample is large, representative, and nearly exhaustive (93%) of the children in public primary schools in Strasbourg, thanks to the consistent data collection over several years.

Dental caries prevalence was high, affecting more than one-third of children in their first year of school, and nearly one-quarter in fifth grade (CM2). A significant decline was observed between 2018 and 2022 which led us to reject the null hypothesis, despite the moderate evolution (33% to 35%, *p* = 0.002). We observed a notable increase in 2020, the year of the COVID-19 lockdown, which will be discussed later.

Inadequate oral hygiene was the strongest predictor of both the presence and number of decayed teeth, with children with inadequate oral hygiene representing only 14% of the total sample but 72% of the children having at least one decayed tooth, and an odd ratio close to 45 in first grade, which decreased in higher grades.

This diminution may reflect the mitigating effect of annual school-based dental check-ups, which facilitate the early detection and management of caries.

The observed decline in caries prevalence with increasing age/grade level may also be partly explained by the transition from primary to mixed and permanent dentition in this age group, as exfoliated carious primary teeth are replaced by healthy permanent teeth.

Despite the overall good oral health status observed (66% of children without any decayed teeth), some inequalities persist, especially when considering the Priority Education Network (REP) status of schools, a well-established indicator of social inequality, or the districts in which schools were localized (unpublished data).

These findings highlight the significant influence of socioeconomic and geographical factors, consistent with previous reports in the literature [11]. The skewed distribution of dental caries in which a small number of children cumulates most caries has long been recognized [12].

Being a boy was also associated with a higher prevalence of dental caries in this study.

This difference may be the consequence of the later adoption of appropriate oral hygiene practices among boys in France [13]. The international literature reports similar caries prevalence between boys and girls during childhood but a higher prevalence among women in adulthood [14].

Our sample of children is slightly smaller in 2020 than during the other years. Children showed a higher prevalence and a greater number of carious lesions.

The COVID-19 pandemic caused lockdowns in 2020. Schools and dental practices were closed for several months. Only emergency dental care was available. This situation delayed access to non-emergency dental treatment. The lockdown periods may have contributed to more sedentary lifestyles, including an increase in snacking and unfavorable dietary habits, which could have further exacerbated the development of dental caries [15].

In 2021 and 2022, a slight increase in caries prevalence was observed compared to 2018–2019 (34–35% vs. 32–33%).

While this increase may be significant due to the large sample size, it may not be clinically relevant. This trend should be analyzed over a longer period. Historical data based on older data (2008–2018) from the same source [16] indicate that caries prevalence remained stable (between 26% and 29%) from 2008 to 2014, exceeding 30% only since 2015, suggesting a gradual deterioration of children’s oral health over recent years.

This situation highlights the importance of targeted prevention and care initiatives for the most affected groups, in addition to population-wide programs.

### 4.2. Limitations of This Study

Data were collected from the medical records obtained by two trained dentists who worked together for years and filled the same standardized medical record. However, inter-examiner reliability was not tested.

This also led to a limitation in the measured variables, especially considering the way carious lesions were recorded without using DMFT or ICDAS and the absence of information about potential confounders such as parental SES, diet, or previous use of dental care.

Inadequate hygiene was determined by the dentists but not clearly defined, probably leading to the extremely strong association we observed between inadequate hygiene and caries prevalence, limiting the external validity of this result. Using a validated tool to measure oral hygiene such as the Oral Hygiene Index or one of its variants [17] would have been more adequate.

Since all Strasbourg children from public schools benefit from the screenings, we were unable to test the specific effect of the prevention program due to the lack of any available control group. Comparison with otherwise similar populations could help measure the real effect of the prevention program on caries prevalence.

Children from private schools are not included. They could present specific profiles considering caries prevalence, socioeconomic status, and other predictors. This limits the generalizability of our results to all Strasbourg children.

### 4.3. Public Health Prevention Policies

The diminution in the number of carious lesions throughout primary school among children in Strasbourg suggests the effectiveness of our local prevention program to treat carious lesions and prevent new ones from appearing. Cost-effectiveness and feasibility studies could be necessary to better evaluate the effectiveness of this program. Depending on the results, similar policies could be implemented at a national level.

School-based interventions are efficient programs to reach all children, including those who are less likely to benefit from programs that require volunteering and autonomy. It could be beneficial to extend this program to target preschool children, considering the already high prevalence and number of carious lesions among children entering their first year of primary school. Extending it to older children in secondary schools could enable longer-term monitoring during a critical period of behavioral development and increasing autonomy among adolescents whose oral health behaviors are more likely to evolve and who have a fully developed permanent dentition.

This study contributes to the identification of effective actions in oral health prevention and promotion. The Strasbourg school-based dental screening program appears to be an efficient local and territorial approach that could be used as a base for the national plan announced by the French government in 2022, which aims to “generalize preventive programs in schools and to introduce daily toothbrushing after meals” as part of a comprehensive oral health strategy [18].

Choosing children as the primary target for preventive strategies is particularly appropriate since dental caries causes irreversible damage and a treated tooth remains structurally weakened, especially when the lesion is detected late. Moreover, inequalities in oral health observed at the age of five often persist in adulthood, supporting the importance of early preventive interventions. Early socioeconomic disparities in oral health have been shown to have consequences extending into the third decade of life, although upward changes in socioeconomic status can lead to improvements [19].

Oral health prevention programs should therefore be combined with broader social and economic inclusion policies to effectively reduce inequalities.

The implementation of adequate oral hygiene and preventive programs remains essential.

The M’T Dents oral health prevention program, announced in November 2005 by the French Minister of Health and Solidarity and recently expanded to cover individuals aged 3 to 25 years annually, includes a major secondary prevention component, comprising fully reimbursed dental check-ups and follow-up care as well as experimental measures targeting high-risk groups. However, the program is voluntary and has so far shown significant limitations in terms of participation rates (38.5% in 2019 which was the highest participation recorded), particularly among populations most at risk [9], while systematic programs in schools such as the one we studied can reach excellent participation rates (93% overall between 2018 and 2022). Cost-effectiveness and outcomes should also be analyzed.

A regular national data collection every few years would allow a better long-term estimation of the oral health of all French children. Implementing school-based interventions following a proactive outreach approach, such as the one implemented in Strasbourg, could help reach most French children.

This survey provides valuable insights for all stakeholders in oral health, particularly considering the apparent deterioration of children’s oral health status. Our findings may be shared with public oral health authorities, including regional public health agencies, which are responsible for implementing prevention programs at the regional level. Finally, this study may contribute to the continuation, adaptation, and development of a comprehensive national policy aimed at improving oral health.

## 5. Conclusions

This study demonstrates that the proportion of children enrolled in Strasbourg’s primary schools presenting with at least one carious lesion decreases progressively throughout their schooling. The same trend is observed for the mean number of carious lesions per child. Despite limitations due to the collection of data from existing medical records, these findings suggest that the prevention program implemented by the City of Strasbourg, which covers all levels of prevention and includes annual dental screenings and follow-up by dentists from the first year of primary/elementary school onward and covers more than 90% of the school children, could be effective in both treating existing caries and preventing the occurrence of new ones, but this needs to be confirmed by studies dedicated to efficiency evaluation.

Socioeconomic status is an important predictor, as children attending priority education zones (REP and REP+) exhibit higher rates of dental caries. This underlines the need to strengthen oral health education programs to raise children’s and families’ awareness of the importance of prevention and early treatment, especially considering the impact of poor oral health on quality of life and social well-being.

Combining all prevention levels from reducing risk to early detection and treatment appears to be the most appropriate strategy for preventing and treating dental caries and could be integrated within broader health equity initiatives. School-based interventions are particularly effective in reaching a large segment of the population and could serve as a model for replication in other cities or for implementation at a national level.

## Figures and Tables

**Table 1:** Proportion of children with at least one decayed tooth and number of decayed teeth (ref: reference level).

	N (%)	Children with at Least One Decayed Tooth	*p*	Number of Decayed Teeth (Mean ± SD)	*p*	Number of Decayed Teeth Among Children with at Least One Decayed Tooth	*p*
**Total**	58,287 (100%)	19,928 (34%)		0.85 ± 1.68		2.5 ± 2.04	
Sex					<0.001		<0.001
Girl	28,590 (49%)	9350 (33%)	ref	0.79 ± 1.61		2.47 ± 1.99	
Boy	29,697 (51%)	10,578 (36%)	<0.001	0.91 ± 2.75		2.60 ± 2.09	
Age (years)					<0.001		<0.001
6	6020 (10%)	2182 (36%)	ref	1.10 ± 2.12		3.09 ± 2. 56	
7	11,258 (19%)	4084 (36%)	0.97	1.05 ± 1.98		2.92 ± 2.33	
8	11,703 (20%)	4339 (37%)	0.28	0.97 ± 1.77		2.66 ± 2.04	
9	11,774 (20%)	4132 (35%)	0.13	0.80 ± 1.53		2.34 ± 1.79	
10	11,745 (20%)	3632 (30%)	<0.001	0.63 ± 1.29		2.10 ± 1.58	
11	5680 (10%)	1526 (26%)	<0.001	0.53 ± 1.19		2.01 ± 1.55	
12	107 (<1%)	33 (30%)	0.25	0.52 ± 1.00		1.75 ± 1.11	
REP status					<0.001		<0.001
Not REP	31,142 (54%)	8265 (27%)	ref	0.61 ± 1.43		2.37 ± 1.94	
REP	13,224 (23%)	5657 (43%)	<0.001	1.15 ± 1.94		2.70 ± 2.16	
REP+	13,543 (23%)	5827 (43%)	<0.001	1.10 ± 1.85		2.63± 2.04	
Grade level					<0.001		<0.001
CP (1st)	11,477 (20%)	4218 (37%)	ref	1.12 ± 2.12		3.09 ± 2.52	
CE1 (2nd)	11,939 (20%)	4449 (37%)	0.45	1.03 ± 1.9		2.82 ± 2.20	
CE2 (3rd)	11,844 (20%)	4307 (36%)	0.50	0.86 ± 1.59		2.44 ± 1.83	
CM1(4th)	11,333 (20%)	3719 (33%)	<0.001	0.70 ± 1.38		2.2 ± 1.64	
CM2 (5th)	11,415 (20%)	3128 (27%)	<0.001	0.52 ± 1.14		1.95 ± 1.44	
Year					<0.001		<0.001
2018	8705 (15%)	2883 (33%)	ref	0.78 ± 1.55		2.42 ± 1.82	
2019	14,804 (25%)	4797 (32%)	0.3	0.83 ± 1.73		2.64 ± 2.17	
2020	9358 (16%)	3454 (37%)	<0.001	0.95 ± 1.82		2.63 ± 2.2	
2021	13,366 (23%)	4556 (34%)	0.12	0.84 ± 1.63		2.54 ± 1.93	
2022	12,054 (21%)	4238 (35%)	0.002	0.85 ± 1.65		2.44 ± 1.99	
Hygiene					<0.001		<0.001
Adequate	47,839 (86%)	13,393 (28%)	ref	0.49 ± 0.99		1.74 ± 1.14	
Inadequate	7922 (14%)	5701 (72%)	<0.001	3.09 ± 2.92		4.30 ± 2.58	

**Table 2:** Mixed-effect logistic regression. Response variable = having at least one decayed tooth.

Variable (ref = Reference Level)	Odds Ratio	Standard Deviation	*p*	95% Confidence Interval	
Sex (ref = Girl)					
Boy	1.17	0.04	<0.001	1.10	1.25
Year (ref = 2018)					
2019	0.84	0.04	<0.001	0.77	0.91
2020	1.00	0.05	0.985	0.91	1.10
2021	0.86	0.04	0.002	0.78	0.95
2022	0.87	0.04	0.004	0.79	0.95
Grade level and hygiene (ref = CP (1st grade) with adequate hygiene)					
Adequate oral hygiene					
CE1 (2nd)	1.10	0.05	0.026	1.01	1.19
CE2 (3rd)	1.15	0.05	0.001	1.06	1.25
CM1(4th)	0.95	0.04	0.295	0.87	1.04
CM2 (5th)	0.69	0.03	<0.001	0.63	0.76
Inadequate oral hygiene					
CP (1st)	44.66	5.20	<0.001	35.55	56.10
CE1 (2nd)	16.88	1.54	<0.001	14.11	20.19
CE2 (3rd)	8.69	0.72	<0.001	7.39	10.22
CM1(4th)	5.05	0.41	<0.001	4.30	5.92
CM2 (5th)	3.15	0.27	<0.001	2.66	3.72
REP Status (ref = non-REP)					
REP	2.48	0.10	<0.001	2.29	2.69
REP+	2.68	0.11	<0.001	2.47	2.91
Residual variance of the random intercept (between individual variances)	2.95	0.10		2.76	3.17

## Data Availability

The datasets presented in this article are not readily available because those data are property of Strasbourg Eurometropole. Requests to access the datasets should be directed to the corresponding author at fernandezdegrado@unistra.fr.
